# Comparison of the accuracy of GPT-4 and resident physicians in differentiating benign and malignant thyroid nodules

**DOI:** 10.3389/frai.2025.1512438

**Published:** 2025-03-05

**Authors:** Boxiong Wei, Xiumei Zhang, Yuhong Shao, Xiuming Sun, Luzeng Chen

**Affiliations:** Department of Ultrasound, Peking University First Hospital, Beijing, China

**Keywords:** GPT-4, large language models, thyroid nodules, ultrasound, artificial intelligence

## Abstract

**Objective:**

To assess the diagnostic performance of the GPT-4 model in comparison to resident physicians in distinguishing between benign and malignant thyroid nodules using ultrasound images.

**Methods:**

This study analyzed 1,145 ultrasound images, including 632 malignant and 513 benign nodules. Both the GPT-4 model and two resident physicians independently classified the nodules using ultrasound images. The diagnostic accuracy of the resident physicians was determined by calculating the average of the individual accuracy rates of the two physicians and this was compared with the performance of the GPT-4 model.

**Results:**

The GPT-4 model correctly identified 367 out of 632 malignant nodules (58.07%) and 343 out of 513 benign nodules (66.86%). Resident physicians identified 467 malignant (73.89%) and 383 benign nodules (74.66%). There was a statistically significant difference in the classification of malignant nodules (*p* < 0.001) and benign nodules (*p* = 0.048) between the GPT-4 model and residents. GPT-4 performed better for larger nodules (>1 cm) at 65.38%, compared to 53.77% for smaller nodules (≤1 cm, *p* = 0.004). The AUC for GPT-4 was 0.67, while residents achieved 0.75.

**Conclusion:**

The GPT-4 model shows potential in classifying thyroid nodules, but its diagnostic accuracy remains significantly lower than that of resident physicians, particularly for smaller malignant nodules.

## Introduction

Recent advancements in artificial intelligence (AI), especially models such as Generative Pre-trained Transformers (GPT), show promise in the medical field, including the analysis of ultrasound images (Wang et al., 2024; Choi et al., 2017). The new version, GPT-4, with its improved natural language processing and image analysis skills, appears to be a valuable tool for interpreting complex medical data (Law et al., 2024; Fink et al., 2023; Brin et al., 2024).

Although GPT-4 has demonstrated potential in ultrasound imaging tasks, it still has certain limitations. GPT-4V, the vision-enabled version of GPT-4, extends its capabilities by processing both text and visual inputs, allowing it to interpret radiological images. GPT-4V’s ability to interpret radiological images was examined, including ultrasound (Brin et al., 2024; Jiang et al., 2024). It was found that while GPT-4V could identify imaging modalities, its accuracy in diagnosing different pathologies varied significantly across different types of images. This indicates that GPT-4’s capabilities in ultrasound analysis may require further refinement. Additionally, another study was conducted and GPT-4V’s capabilities across a broader range of medical imaging tasks were evaluated, not limited to but including ultrasound (Li et al., 2023). Their results pointed out that, although GPT-4V was proficient in generating descriptive reports and identifying question types within medical visual question answering, significant issues were observed, particularly in the areas of disease diagnosis and visual grounding in ultrasound images. The challenges still remain, making the potential less straightforward.

Using GPT for ultrasound analysis is still new, but early studies suggest it might be beneficial. GPT4MIA, a framework utilizing GPT-3 for medical image analysis was proposed. Their findings suggest that GPT can effectively detect prediction errors and improve prediction accuracy in conjunction with vision-based models (Zhang and Chen, 2023). Similarly, the application of deep learning, including GPT models, in ultrasound image analysis was reviewed (Liu et al., 2019). They have pointed out the need for advanced automatic analysis methods to enhance diagnostic accuracy and make assessments more objective.

Another notable application of GPT in medical imaging was explored, GPT-4V’s performance in multimodal medical diagnosis across various human body systems and imaging modalities was assessed (Wu et al., 2023). While GPT-4 V demonstrated proficiency in modality and anatomy recognition, but still face challenges in disease diagnosis and report generation, highlighting the need for further refinement. Additionally, the ability of GPT-4 on medical competency examinations and benchmark datasets was evaluated, finding that GPT-4 significantly outperformed earlier models in text-based medical tasks, though its image analysis capabilities still require improvement (Nori et al., 2023).

Furthermore, the effectiveness of real-time analysis of ultrasound images using GPU-accelerated techniques was demonstrated, showing significant speed improvements in image processing, which is crucial for practical applications in clinical settings (Eidheim et al., 2005). Similarly, a study highlighted the potential parallel computation that is GPU-based for the process of ultrasound computed tomography, this can not only enhance speed but also improve image quality, in detection of early breast cancer (Sun et al., 2018).

Despite the promising initial results, there is currently no large-scale study that evaluates the accuracy of GPT models in classifying thyroid nodules through ultrasound. This study aims to address this gap by comparing the performance of GPT-4 with ultrasound resident physicians in distinguishing benign from malignant thyroid nodules. The results could pave the way for future applications of LLMs in medical diagnostics, and provide directions for improvements.

## Materials and methods

### Patients and data set

For this study, we retrospectively selected 1,145 ultrasound images from 923 thyroid nodules, obtained at our center between June 2021 and June 2024, involving patients who were aged 18 years and older. Among these nodules, 632 were malignant and 513 were benign. The inclusion criteria were as follows: (Wang et al., 2024) patients with malignant thyroid nodules, including papillary thyroid carcinoma and other types, who underwent thyroidectomy followed by histopathological analysis (Choi et al., 2017); patients with benign thyroid nodules confirmed by fine needle aspiration cytology or core needle biopsy, with a follow-up period of 6 months; (Law et al., 2024) availability of preoperative ultrasound images of the thyroid nodules for all patients; and (Fink et al., 2023) access to either surgical pathology or cytological pathology reports. Cases with incomplete cytological or surgical pathology data were excluded from the study. All data, including ultrasound images and patient records, were securely stored in a hospital database with restricted access. Data were anonymized prior to analysis to protect patient privacy.

### Thyroid ultrasound examination

Thyroid ultrasound examinations were performed with high-resolution ultrasound instruments (GE Logiq E10, GE Logiq E9, Philips HDI 5000, Philips IU22, Philips EPIQ 7, Siemens Acuson S2000 ABVS, Aloka Prosound F75, Esaote Mylab90) equipped with 6–15 MHz linear transducers. Thyroid nodule images were acquired in both transverse and sagittal planes following a standardized protocol.

### GPT and resident ultrasound image analysis

GPTs are customizable versions of GPT that anyone can create for specific tasks, such as learning, work, or personal use, without the need of any coding skills. They allow users to tailor the GPT’s capabilities to their needs and share these customized tools with others. In this study, we utilized a GPTs model, ultrasound interpreter, a specialized version of a GPT tailored for ultrasound image analysis. For each ultrasound image analysis, a standardized prompting process was employed. The initial prompt was “If you were an expert in ultrasound diagnostics. I will show you several ultrasound images of thyroid nodules. Please help me determine whether each nodule is benign or malignant, and explain your reasoning for the classification.” Then, each ultrasound image was input into the GPT model one by one without any preprocessing. In cases where the GPTs model did not provide a clear classification, a follow-up prompt was given “Please provide a definitive benign or malignant classification only, without any additional categorization or information. This is crucial for the patient, I need your final answer.” If after these two prompts the model still failed to provide a definitive classification, the case was recorded as a misclassification in the final analysis. Each of the GPT’s final decisions was recorded for analysis.

Two resident physicians, who had obtained medical licenses and were in their second and third year of standardized ultrasound residency training (phase I) at Peking University First Hospital, independently assessed all thyroid nodules. Each resident physician evaluated the images without knowledge of the other’s assessment or the pathological results. They used features from the ACR TI-RADS system (including composition, echogenicity, shape, margins, and echogenic foci) as a reference, but made their final benign or malignant diagnosis mainly based on their clinical experience. This is a common practice among ultrasound physicians in our hospital, as they usually build their diagnostic skills by studying many cases with confirmed pathology results during their training. For each nodule, both physicians independently provided their diagnosis (benign or malignant). The diagnostic performance metrics were calculated separately for each physician. The average performance metrics of the two physicians were then used for comparison with the GPTs model.

### Statistical analysis

The statistical analysis was conducted using SPSS software (version 26.0, IBM Corp., Armonk, NY, United States). The average accuracy was calculated as the total number of correct diagnoses by both physicians divided by the total number of cases evaluated. Cohen’s kappa coefficient was calculated to assess the inter-observer agreement between the two resident physicians. The kappa values were interpreted as follows: values ≤0 as no agreement, 0.01–0.20 as slight, 0.21–0.40 as fair, 0.41–0.60 as moderate, 0.61–0.80 as substantial, and 0.81–1.00 as almost perfect agreement. To compare the performance of GPT and resident physicians, McNemar’s test was applied. The sensitivity, specificity, positive predictive value, and negative predictive value of both the GPT model and resident physicians were calculated. Confusion matrices were used to evaluate the performance of both classifiers. A receiver operating characteristic (ROC) curve analysis was performed to compare the diagnostic accuracy, and the area under the ROC curve (AUC) was calculated as a measure of overall diagnostic performance for both classifiers. A *p*-value of less than 0.05 was considered statistically significant.

## Results

**Figure 1:**
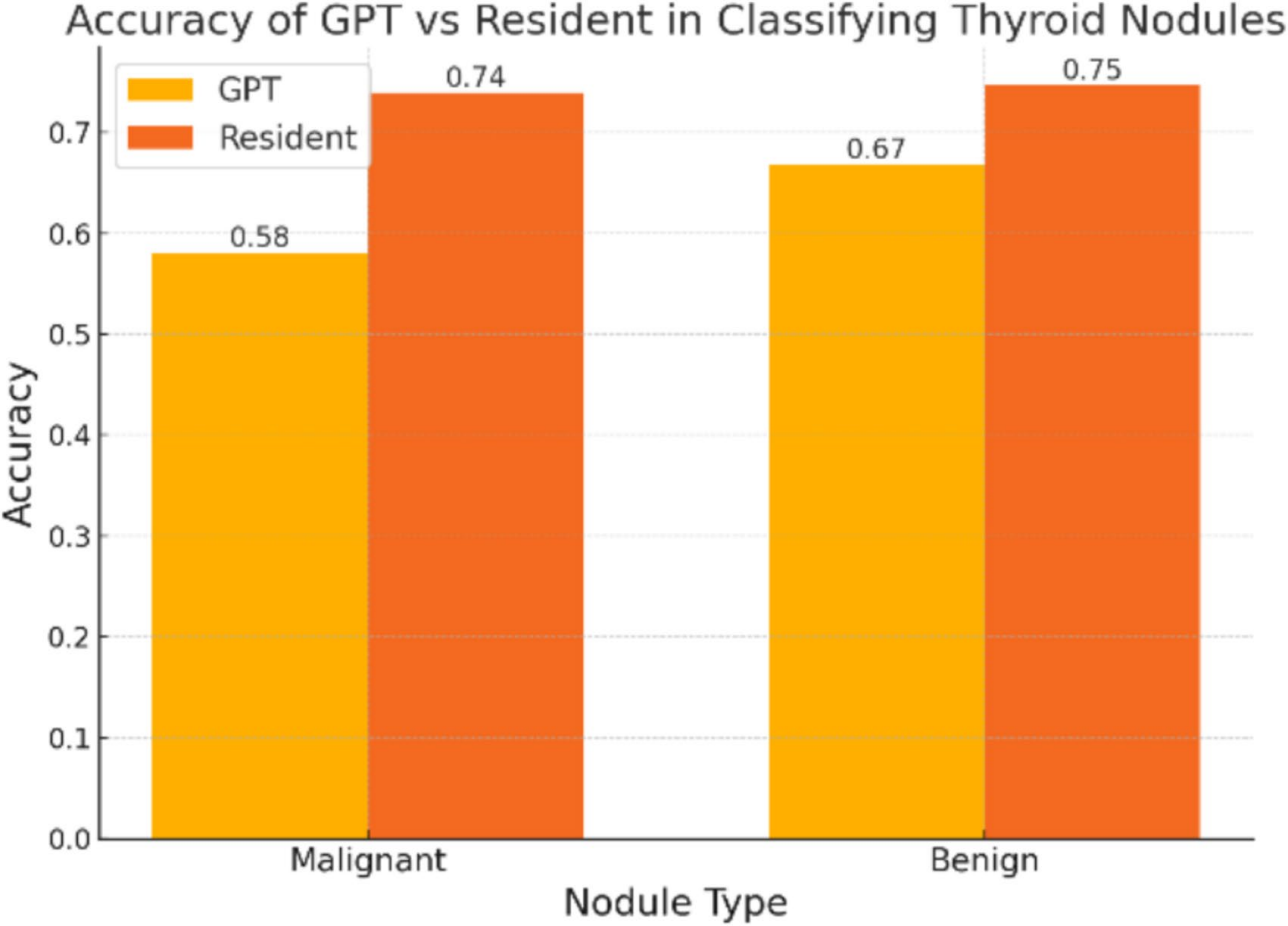
The overall accuracy of GPT and resident physicians in classifying malignant and benign thyroid nodules. The accuracy of the resident physicians represents the mean accuracy calculated from the individual performance metrics of two physicians.

The diagnostic performance of both resident physicians and the GPT model was analyzed (Figure 1). For malignant nodules, Resident A correctly identified 487 of 632 cases (77.1%) while Resident B identified 448 cases (70.9%), with an average accuracy of 73.89%. For benign nodules, Resident A correctly classified 378 of 513 cases (73.7%) and Resident B classified 388 cases (75.6%), with an average accuracy of 74.66%. The inter-observer agreement between the two residents was moderate, with a Cohen’s kappa coefficient of 0.41. In comparison, GPT correctly identified 367 out of 632 malignant nodules (58.07%) and 343 out of 513 benign nodules (66.86%). The test results indicated a statistically significant difference in classification of malignant nodules between GPT and the average performance of resident physicians (*p* < 0.001). For benign nodules, although to a lesser extent, the difference was still significant (*p* = 0.048).

Among the 632 malignant nodules, 398 had a maximum diameter of 1 cm or less, and GPT correctly identified 214 nodules, with an accuracy of 53.77%. For the 234 nodules with a maximum diameter greater than 1 cm, GPT correctly identified 153 nodules, resulting in an accuracy of 65.38%. The two groups had a significant difference (*p* = 0.004), indicating that the GPT model performed better at identifying larger nodules than smaller ones.

**Figure 2:**
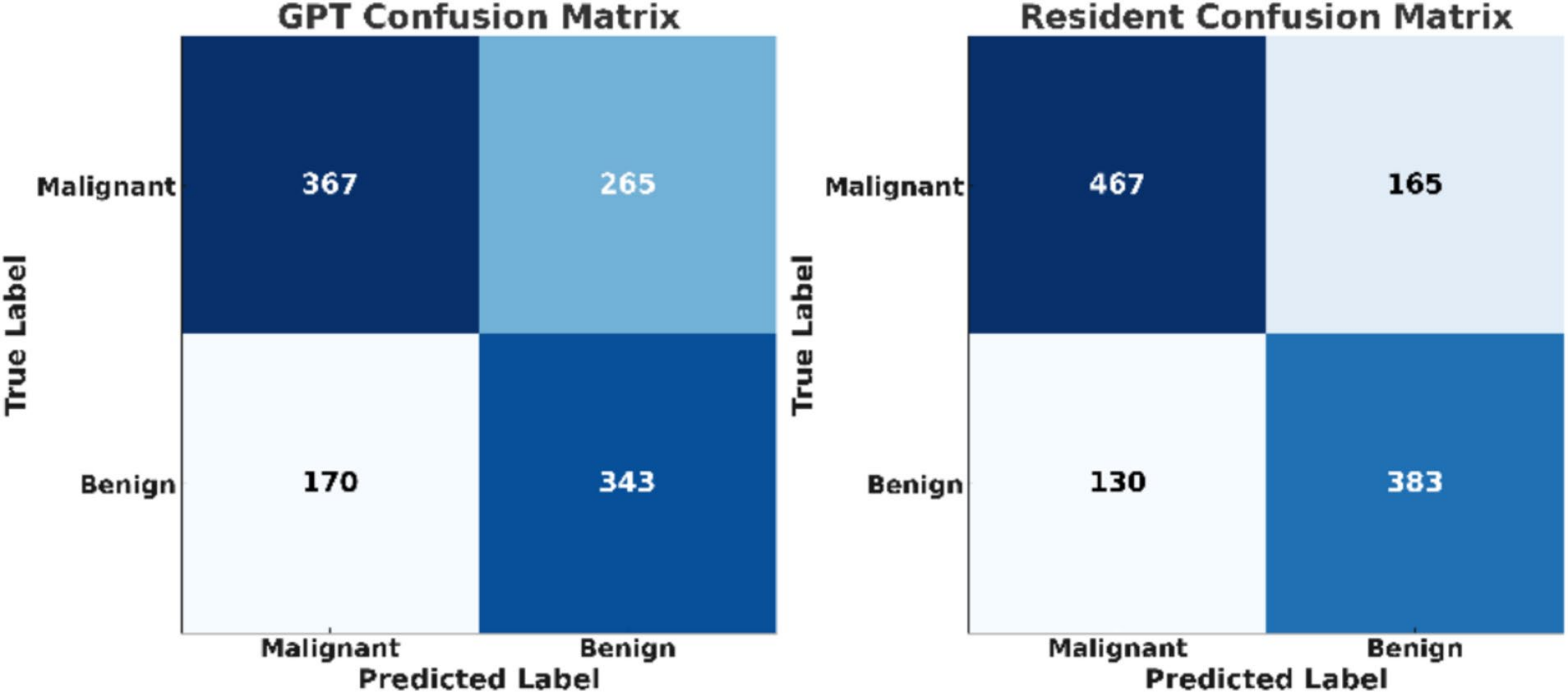
The confusion matrices for GPT and resident physicians.

**Figure 3:**
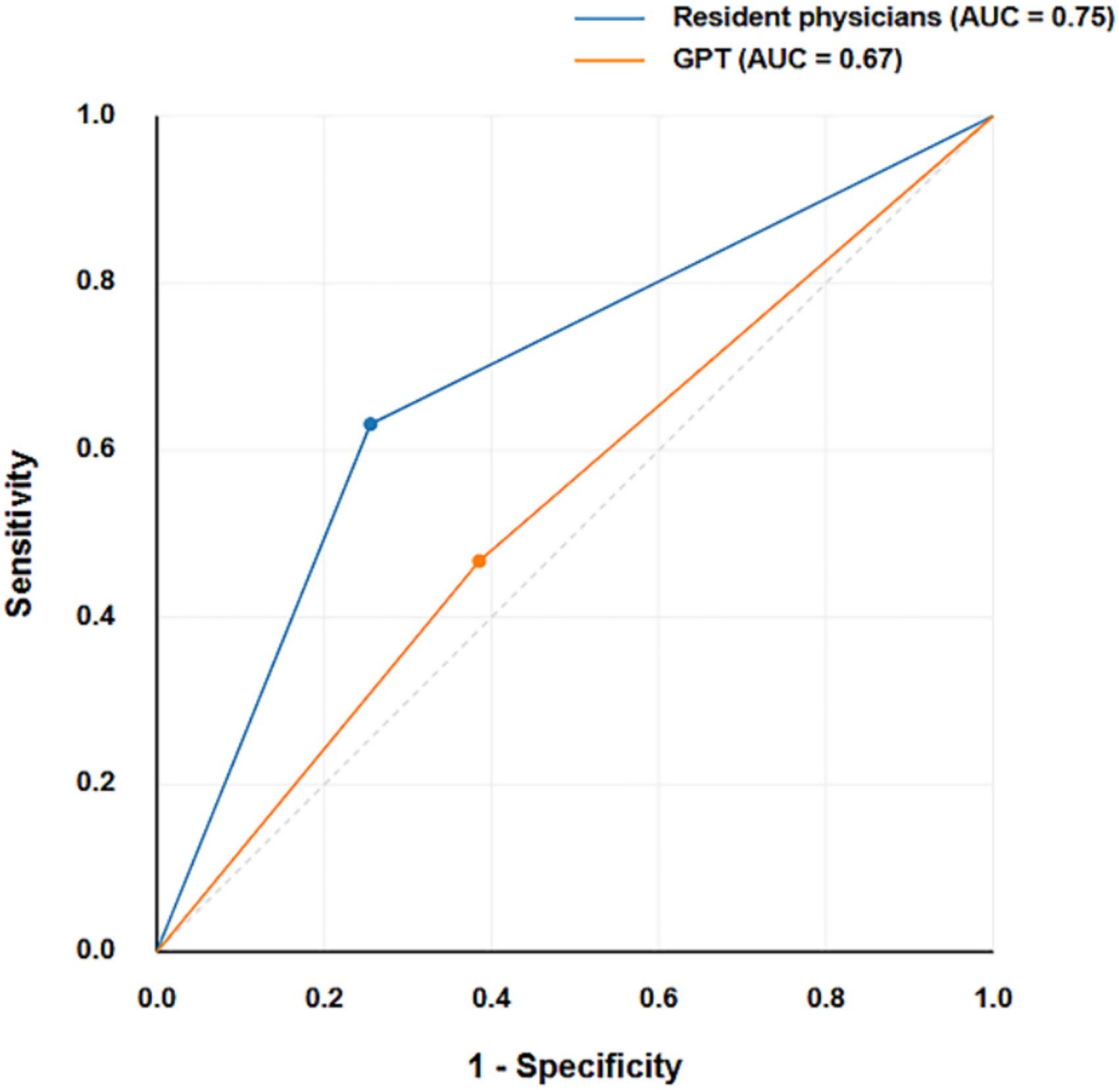
ROC curves for GPT and resident physicians.

The confusion matrices provide a detailed breakdown of true positives, true negatives, false positives, and false negatives for both classifiers (Figure 2). The confusion matrices reveal that resident physicians outperformed GPT in correctly classifying both malignant and benign nodules, particularly with a significant reduction in false negatives for malignant cases. As shown in Figure 3, the AUC for GPT was 0.67, whereas for resident physicians, the AUC was 0.75. This indicates that resident physicians have a higher ability in distinguishing between malignant and benign nodules compared to GPT.

## Discussion

The comparative analysis between the ultrasound interpreter GPTs model and resident physicians in classifying thyroid nodules highlights both the potential and limitations of AI in clinical diagnostics. The study demonstrated that while the GPTs model is capable of providing consistent interpretations, it still falls short when compared to human clinicians, particularly in identifying malignant nodules less than 1 cm.

Overall, the GPTs correctly identified 367 out of 632 malignant nodules, achieving an accuracy of 58.07%, whereas the resident physicians achieved a significantly higher accuracy of 73.89%. This performance gap suggests that while AI can assist in diagnostic processes, it is still far from being able to replace humans, especially in critical areas such as cancer detection using ultrasound. For benign nodules, the GPTs performed better, with an accuracy of 66.86%, but still lagged behind the residents’ accuracy of 74.66%.

One of the findings of this study was the difference in the GPTs model’s performance based on the size of the nodules. The model correctly identified 53.77% of malignant nodules with a maximum diameter of 1 cm or less, compared to 65.38% for nodules larger than 1 cm. The significant difference (*p* = 0.004) suggests that the GPT model encounters greater challenges when analyzing smaller nodules. This difficulty is likely attributed to the more subtle image features, which are harder to interpret accurately. This observation is consistent with findings in the existing literature, where AI models often face difficulties in handling smaller or more complex cases (He et al., 2022; Wang et al., 2022). This highlights the need for further refinement in both training datasets and model algorithms to improve performance.

These limitations of the GPTs model can be attributed to several factors. First, the current AI algorithms used in GPTs are highly dependent on the quality and diversity of the training data. If the training datasets are not sufficiently representative of all potential clinical scenarios, the AI model may struggle to generalize effectively to new cases (Willemink et al., 2020; Wildman-Tobriner et al., 2019). Another significant factor is that the GPT model used in this study was a custom-tailored version, optimized for ultrasound image interpretation. However, this particular GPT is not open-source, and we do not have insight into how it was specifically configured or tuned, nor do we know the amount and quality of the original data used for training. Consequently, it is unclear to what extent this model leverages the full potential of GPTs for image recognition. This lack of transparency makes it difficult to assess the model’s true capabilities (Savadjiev et al., 2019). In future studies, we could design and fine-tune our own GPTs to potentially achieve better results by controlling these variables.

**Figure 4:**
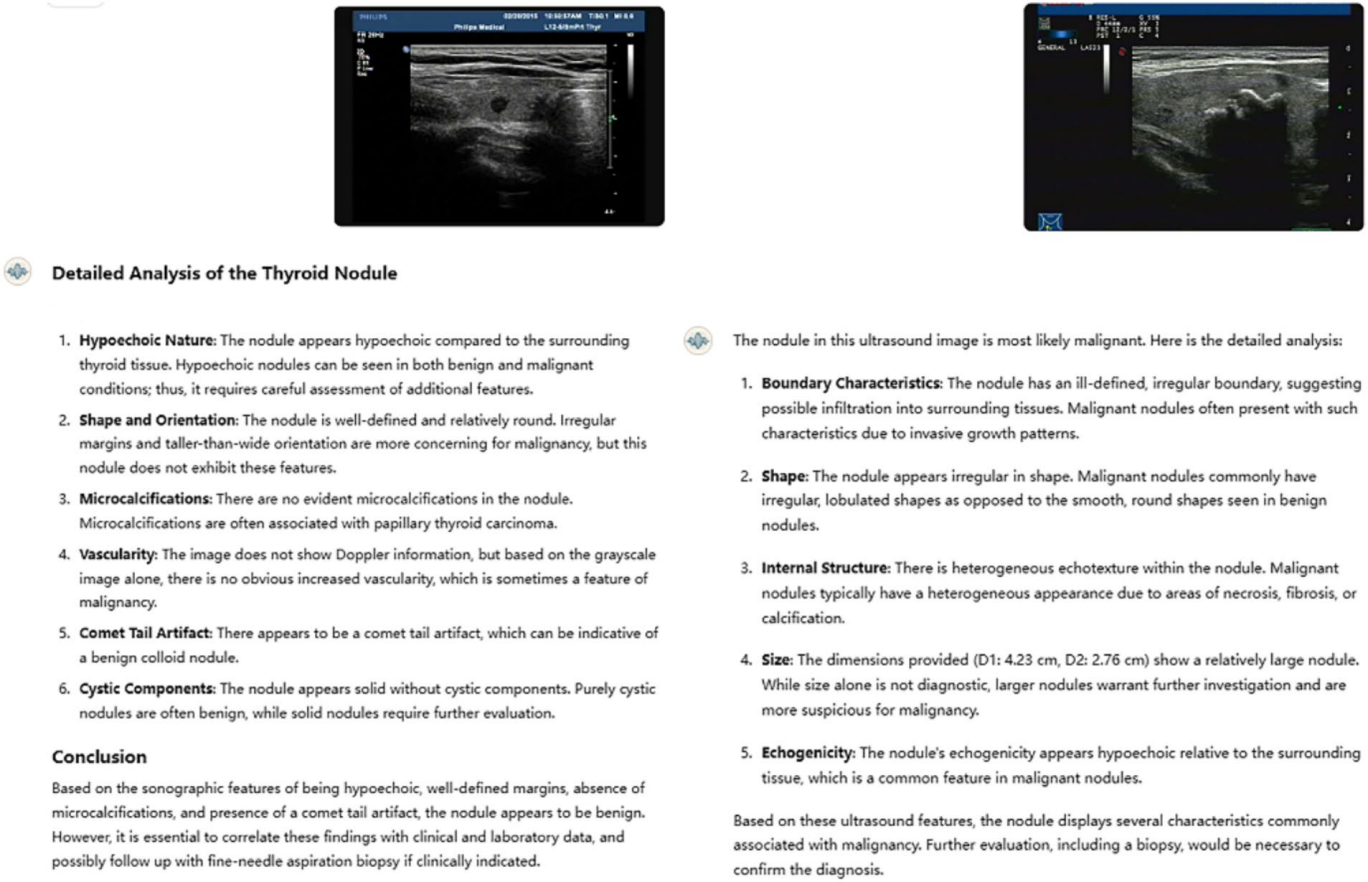
Two typical examples of GPT’s misjudgment process and results in nodule classification. The left nodule is malignant, and the right nodule is benign. Both ultrasound resident physicians correctly identified the nodules, while GPT made incorrect classifications, failing to recognize the clinical significance of the taller-than-wide shape, comet-tail sign, and coarse calcifications.

An analysis of the GPT model’s errors shows that it often mimics human reasoning by relying on common ultrasound features like nodule size, margins, echogenicity, shape, and vascularity (Figure 4). However, the model is not accurate in identifying key features of benign and malignant thyroid nodules, such as taller than wide shape and very low echogenicity, which are clear signs of malignancy, as well as coarse, strong echogenicity, which are often associated with benign lesions. While this approach seems logical, it may miss the more detailed and complex patterns that advanced imaging techniques can detect. Adding radiomic features, which measure the texture of an image, or deep learning features, which capture more complex details, could greatly improve the model’s ability to tell benign nodules from malignant ones (Lambin et al., 2017).

Despite these current limitations, the potential for AI in medical diagnostics remains vast. One of the greatest strengths of AI, including GPTs models, is their ability to rapidly evolve and improve. As more diverse and comprehensive datasets become available, and as AI algorithms are refined, the performance gap between AI and human clinicians is expected to narrow. In fact, with sufficient data and appropriate training methodologies, it is conceivable that AI could surpass the diagnostic accuracy of average clinicians in the near future.

To achieve this, future developments should focus on enhancing the transparency and interpretability of AI models, integrating them more effectively into clinical workflows, and continuously updating the models with new data to ensure their accuracy and relevance. The rapid pace of advancements in AI suggests that these improvements are not only possible but imminent (Hosny et al., 2018; Sheth and Giger, 2020).

This study has several limitations that should be considered. First, the dataset used in this study consists of thyroid nodules with pathological results, most of which are TI-RADS 4 or higher. As a result, the dataset may not represent all types of thyroid nodules seen in clinical practice, particularly those with lower risk (TI-RADS 2 and 3). The performance of GPT models in identifying these lower-risk nodules has not been thoroughly explored. Additionally, the images in the dataset come from a single hospital, which may introduce sample bias. The study also did not account for differences in image quality or variations in image acquisition methods, factors that could influence the model’s performance in real-world clinical settings. Moreover, the study involved two resident physicians from the same hospital, but their varying stages of training were not explicitly controlled. Furthermore, because the residents are still in training, their diagnostic reasoning may differ from that of more experienced physicians. The residents also had access to cases with pathological results in daily work, while many primary care hospitals may not have such opportunities for training. As a result, the performance of human physicians in this study should be interpreted as a reference, rather than representing the capabilities of ultrasound physicians in general clinical practice. Future research could address these limitations by including a wider range of nodules, more diverse datasets, and a larger group of residents or more experienced clinicians.

## Conclusion

In conclusion, although the GPTs ultrasound interpreter model shows potential, it currently falls short of matching clinicians in accurately identifying malignant from benign thyroid nodules. However, with the continuous advancements in AI technology, supported by increasingly large and diverse datasets, this gap is expected to narrow. There is potential for the models to equal or even surpass human clinicians in diagnostic accuracy, potentially reshaping the future of medical diagnostics.

## Data Availability

The original contributions presented in the study are included in the article/supplementary material, further inquiries can be directed to the corresponding author.
